# Machine learning model combining features from algorithms with different analytical methodologies to detect laboratory-event-related adverse drug reaction signals

**DOI:** 10.1371/journal.pone.0207749

**Published:** 2018-11-21

**Authors:** Eugene Jeong, Namgi Park, Young Choi, Rae Woong Park, Dukyong Yoon

**Affiliations:** 1 Department of Biomedical Informatics, Ajou University School of Medicine, Suwon, Gyeonggi-do, Republic of Korea; 2 College of Pharmacy, Ewha Womans University, Seoul, Republic of Korea; 3 Department of Biomedical Sciences, Ajou University Graduate School of Medicine, Suwon, Gyeonggi-do, Republic of Korea; University of Oxford, UNITED KINGDOM

## Abstract

**Background:**

The importance of identifying and evaluating adverse drug reactions (ADRs) has been widely recognized. Many studies have developed algorithms for ADR signal detection using electronic health record (EHR) data. In this study, we propose a machine learning (ML) model that enables accurate ADR signal detection by integrating features from existing algorithms based on inpatient EHR laboratory results.

**Materials and methods:**

To construct an ADR reference dataset, we extracted known drug–laboratory event pairs represented by a laboratory test from the EU-SPC and SIDER databases. All possible drug–laboratory event pairs, except known ones, are considered unknown. To detect a known drug–laboratory event pair, three existing algorithms—CERT, CLEAR, and PACE—were applied to 21-year inpatient EHR data. We also constructed ML models (based on random forest, L1 regularized logistic regression, support vector machine, and a neural network) that use the intermediate products of the CERT, CLEAR, and PACE algorithms as inputs and determine whether a drug–laboratory event pair is associated. For performance comparison, we evaluated the sensitivity, specificity, positive predictive value (PPV), negative predictive value (NPV), F1-measure, and area under receiver operating characteristic (AUROC).

**Results:**

All measures of ML models outperformed those of existing algorithms with sensitivity of 0.593–0.793, specificity of 0.619–0.796, NPV of 0.645–0.727, PPV of 0.680–0.777, F1-measure of 0.629–0.709, and AUROC of 0.737–0.816. Features related to change or distribution of shape were considered important for detecting ADR signals.

**Conclusions:**

Improved performance of ML models indicated that applying our model to EHR data is feasible and promising for detecting more accurate and comprehensive ADR signals.

## Introduction

Pharmacovigilance refers to the processes used for detecting adverse drug reactions (ADRs) or other drug-related problems to prevent them.[1] It is divided into pre-approval stage activities (during phase I–III of clinical trials) and post-approval stage activities (phase IV clinical trial or post-market surveillance).[2] Because a complete ADR profile cannot be fully established through clinical trials and ADRs can incur very high costs (estimated to be $75 billion annually in the US alone[2, 3]), continuous monitoring of safety even after a drug is marketed is essential. Post-market surveillance is aimed at establishing the ADR profile of a certain drug, and it needs to be distinguished from other approaches[4–6] aimed at detecting individual clinical adverse events occurring in daily practice.

Recently, many studies have used electronic health record (EHR) data for ADR signal (i.e., information suggesting a new ADR) detection for post-market surveillance because of the large-scale collection of computerized clinical data in EHRs.[7–12] EHR data include a longitudinal electronic record of a patient’s condition, such as diagnosis, laboratory test results, and radiology test results, along with the drugs the patient is exposed to. Thus, EHR data are a useful source of information on the association of drugs with certain ADRs. Among the diverse information in EHRs, laboratory test results are relatively more objective and quantitative than other descriptive records written by healthcare providers, although they may be influenced by factors such as equipment and protocols.[13] Therefore, laboratory test records can make large-scale analysis easy with an automatic algorithm.[12, 14]

We have published three algorithms to detect ADR signals using laboratory test results in EHRs based on different analytical methodologies.[7, 8, 10] The Comparison of Extreme Laboratory Test results (CERT) algorithm compares laboratory test results before and after the patient’s drug exposure.[7] Therefore, there is no bias due to patient characteristics; however, the results can be biased by the time-dependent covariate because the patient’s condition can change during treatment. To solve this problem, we developed the Comparison of Extreme Abnormality Ratio (CLEAR) algorithm.[8] This algorithm compares the frequency of an event in drug-exposed patients with that in matched nonexposed controls. In this algorithm, time-dependent covariates can be corrected. However, the bias due to differences in patients cannot be corrected perfectly. Another problem was confounding by indication in which the drug used for treatment was incorrectly detected as an ADR signal. To solve this problem, we proposed the Prescription pattern Around Clinical Event (PACE) algorithm.[10] This algorithm analyzes the drug prescribing pattern before and after the clinical event to provide a prescription change index (PCI) to distinguish between the treatment and ADR.

As described above, owing to the advantages and disadvantages of their analytical methodologies, none of the algorithms can completely replace the others. At the same time, each algorithm can compensate for the weakness of the others. However, no studies have focused on how to combine these algorithms. Machine learning (ML) algorithms can find optimized weights for input values. Therefore, we applied ML to integrate the results from existing algorithms and derive a single, more accurate result.

This study aims to develop a more accurate ADR signal detection algorithm for post-market surveillance using EHR data by integrating the results of existing ADR detection algorithms using ML models. We compared the performance of existing methods and that of the ML models combining the results of existing methods when they are applied to 21-year EMR data from a tertiary teaching hospital.

## Materials and methods

This study was approved and informed consent was waived by the Ajou University Hospital Institutional Review Board [IRB No. AJIRB-MED-MDB-17-185]. Only deidentified data were used and analyzed retrospectively.

### Clinical data source

**Fig 1:**
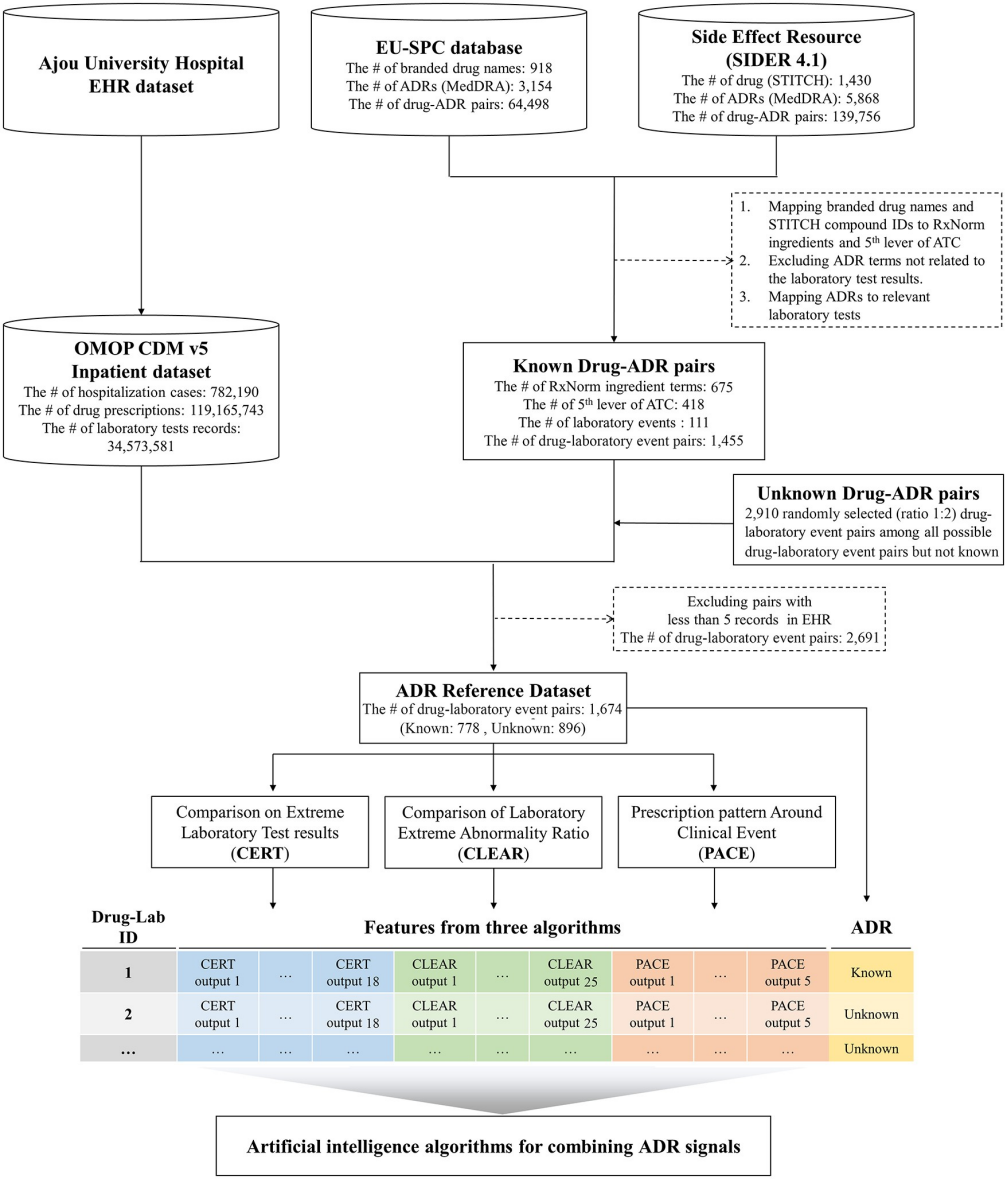
Study schematic. Existing CERT, CLEAR, and PACE algorithms are applied to the Ajou University Hospital EHR dataset to extract features for ML algorithms for combining ADR signals. EU-SPC and SIDER databases are used to define the ADR reference dataset.

We used EHR data for the entire hospitalization period for 475,417 patients treated in Ajou University Hospital from June 1, 1994, to April 15, 2015 (Fig 1). The clinical data used herein were those transformed to the Observational Medical Outcomes Partnership (OMOP) common data model (CDM) of the Observational Health Data Science and Informatics (OHDSI) consortium for research purposes.[15] The database included 119,165,743 drug prescription and 34,573,581 laboratory test records (53 distinct laboratory tests) from 782,190 hospitalization cases. The average and standard deviation of the observational period (from admission to discharge) was 8.6 and 16.1 days, respectively.

### ADR databases

To construct the ADR reference dataset, drug–ADR associations were collected from the widely used European Union Adverse Drug Reactions from Summary of Product Characteristics (EU-SPC) database that was constructed by the PROTECT collaborative and the Side Effect Resource (SIDER 4.1).[16] The EU-SPC database includes all ADRs listed in the SPC of drugs authorized in the EU, and SIDER 4.1 provides information on drugs, adverse events, and indications mined from FDA package inserts and other drug documentation. A total of 918 drugs, 3,154 ADRs, and 64,498 drug–ADR pairs are available in the EU-SPC database. SIDER 4.1 contains data on 1,430 drugs, 5,880 ADRs, and 140,064 drug–ADR pairs.

### Generating ADR reference dataset from ADR databases

#### Known ADR–laboratory event pairs

The drug terminologies used in the EU-SPC and SIDER 4.1 databases were STITCH compound IDs and branded names, respectively; therefore, it is critical to map from the drug terms used in each database to RxNorm, which is the standard terminology for drugs in OMOP CDM. By using the mapping table constructed by LAERTES[17] and the OMOP Standard Vocabularies, we primarily mapped the branded drug names and the STITCH compound ID to RxNorm ingredients. Two clinical experts reviewed all assigned RxNorm concepts and manually mapped terms that could not be mapped through mapping tables (S1 Table). The number of distinct RxNorm concepts for drugs was 675.

Both ADR databases use the ADR names from the MedDRA dictionary and provide access to preferred and lower-level terms. We extracted 6,053 unique MedDRA ADR terms from the databases. Among the MedDRA ADR terms in combined ADR databases, only those associated with abnormal laboratory test results were chosen to analyze drug–laboratory events in the EHRs. We filtered 6,053 distinct ADR terms down to 249 (~4%); these included the words “increase,” “decrease,” “hyper-,” “hypo-,” “prolonged,” or “shortened” that represent most laboratory abnormality cases. However, this does not mean that our study covers only ~4% of terms because the ADR database contains various overlapping and closely related ADR terms. For example, although “hepatotoxicity” was not included in this study, it can be covered by “AST increased” or “ALT increased.”[7] Lastly, we developed a mapping table linking between ADR terms and adverse laboratory events from EHR data to use laboratory test results as surrogate markers. For example, we mapped the ADR terms “Hyperglycemia,” “Blood glucose increased,” and “Blood sugar increased” to the laboratory event “Glucose (AC) increased.” Two clinical experts removed duplicated ADR terms, as shown in the example above, thereby mapping the 249 selected ADR terms to 111 laboratory events.

Finally, from among all known drug-ADR pairs in the EU-SPC and SIDER 4.1 databases, 1,455 drug-laboratory event pairs (consisting of 675 RxNorm concepts and 111 laboratory events) were selected as known drug–laboratory event pairs.

#### Datasets for ML model development and performance test

All possible combinations between 675 distinct RxNorm concepts and 111 laboratory events were matched, and 74,925 drug–laboratory event pairs were generated. We considered the drug–laboratory event pairs recorded in ADR databases as positives (= 1) and pairs were not present in the ADR databases as nonpositive (= 0).

To balance between positive and nonpositive data, we randomly selected two times as many pairs among all nonpositive data as those among positive data and then excluded drug–laboratory event pairs with less than five records in EHR data. The final ADR reference dataset included 778 known (positive) drug–laboratory event and 896 unknown (nonpositive) pairs. S2 Table lists all 1,674 drug–laboratory event pairs. The dataset was then divided into two subsets: dataset for ML model development and dataset for performance evaluation (test dataset) in 70:30 ratio.

### Feature extraction from ADR algorithms based on different analytical methodologies

Three different algorithms based on different analytical methodologies were applied to the EHR data: CERT, CLEAR, and PACE.[7, 8, 10] The final output and intermediate products (values calculated from input and used for calculating final output, like descriptive statistics or measures on shape of data distribution) of each algorithm were extracted for use as features for the ML algorithms (Table 1).

**Table 1:** Features derived from three ADR signal detection algorithms.

Algorithm	Features
CERT	1. Average of laboratory test results before drug exposure 2. Median of laboratory test results before drug exposure 3. Standard deviation of laboratory test results before drug exposure 4. Kurtosis of laboratory test results before drug exposure 5. Skewness of laboratory test results before drug exposure 6. Number of patients whose laboratory results are within normal range before drug exposure 7. Number of patients whose laboratory results are out of normal range before drug exposure 8. Average of laboratory test results after drug exposure 9. Median of laboratory test results after drug exposure 10. Standard deviation of laboratory test results after drug exposure 11. Kurtosis of laboratory test results after drug exposure 12. Skewness of laboratory test results after drug exposure 13. Number of patients whose laboratory results are within normal range after drug exposure 14. number of patients whose laboratory results are out of normal range after drug exposure 15. *p*-value from paired *t*-test or Wilcoxon signed-rank test 16. *p*-value from McNemar’s test or McNemar’s exact test 17. Absolute percentage change in a lab value pre and post exposure 18. Percentage of patients whose lab results changed from normal to abnormal after exposure
CLEAR	19. Average of laboratory test results in a risk group 20. Median of laboratory test results in a risk group 21. Standard deviation of laboratory test results in a risk group 22. Kurtosis of laboratory test results in a risk group 23. Skewness of laboratory test results in a risk group 24. Number of patients in a risk group whose laboratory test results were within normal range 25. Number of patients in a risk group whose laboratory test results were out of normal range 26. Absolute percentage change in average of laboratory test results pre and post exposure in a risk group 27. Percentage of patients whose lab results changed from normal to abnormal after exposure 28. Average of laboratory test results in a control group 29. Median of laboratory test results in a control group 30. Standard deviation of laboratory test results in a control group 31. Kurtosis of laboratory test results in a control group 32. Skewness of laboratory test results in a control group 33. Number of patients in a control group whose laboratory results were within normal range 34. Number of patients in a control group whose laboratory results were out of normal range 35. Absolute percentage change in average of laboratory test results pre and post exposure in a control group 36. Percentage of patients whose lab results changed from normal to abnormal after exposure 37. *p*-value from conditional logistic regression 38. *p*-value from Fisher’s exact test 39. Yule’s Q– 1.96SE 40. PRR– 1.96SE 41. ROR– 1.96SE 42. IC– 2SD 43. EB05
PACE	44. Prescription counts on three days before the clinical event occurred 45. Prescription counts on two days before the clinical event occurred 46. Prescription counts on the date of the clinical event 47. Prescription counts a day after the clinical event 48. Prescription change index

SE, standard error; PRR, proportional reporting ratio; ROR, proportional odds ratio; IC, information component; SD, standard deviation; EB05, the lower bound of the 90% confidence interval for the Empiric Bayes Geometric Mean (EBGM)

#### CERT algorithm

In the original version of the CERT algorithm, the maximum or minimum laboratory results of paired observations (before and after drug exposure) were compared by a paired t‐test for each drug–laboratory event pair. The same extreme pairs were compared for the differences in the occurrence of abnormal laboratory test results (results that fell below or above the reference range) before and after medication by McNemar’s test. The pair was considered a positive signal when the paired t‐test or McNemar’s test was significant (p < 0.05). When we used the CERT algorithm to extract the input features of our model, we additionally conducted tests that were not considered in the original article to obtain more accurate results. If its sample size is above 30 or the laboratory results of paired observations follow a normal distribution, a paired t-test was used. If not, Wilcoxon’s signed-rank test was used. To account for multiple testing, the Bonferroni correction was used to adjust the *p*-value.

#### CLEAR algorithm

The CLEAR algorithm searches for associations between drug exposure and laboratory test abnormalities by comparing drug-exposed patients and matching nonexposed controls.[8] The odds ratio (OR) and 95% confidence interval for the association between each drug and an abnormal laboratory result were evaluated by conditional logistic regression in the CLEAR algorithm. A confidence interval with lower limit > 1.0 was considered a positive signal.

#### PACE algorithm

Three prescription patterns (discontinuation, intervention, and maintenance pattern) were defined in the PACE algorithm according to the PCI. PCI represents the level of decrease or increase in the prescription number after the event occurred. While the discontinuation pattern was defined as PCI ≤ 0.667 (1.0/1.5), >1.500 was defined as an intervention pattern, and the PCI between two cut-offs was classified as a maintenance pattern.

### Development of ML models for combining features from different algorithms

#### Data preprocessing

We normalized the laboratory test result values from the CERT and CLEAR algorithms using the min-max scaling method before descriptive statistics were calculated to adjust different scales according to different types of laboratory tests for being used as a single feature.[18] We extracted 18, 25, and 5 features from CERT, CLEAR, and PACE, respectively, and all these 48 features were standardized over the entire dataset so that each feature has mean = 0 and SD = 1, and they were used as inputs to the ML models.

#### Hyperparameter optimization

We built ML models using L1 regularized logistic regression, random forest, support vector machines (SVMs), and neural network. To obtain the optimal values of model parameters, we used the GridSearchCV function from the scikit-learn library in the Python programming language to explore all parameter combinations and choose the parameters that result in the best model using tenfold cross-validation. Parameter combinations were tested, and the best parameters we chose for each model are as follows. For the L1 regularized logistic regression model, the combinations of penalty∈[‘l1’,‘l2’] and C∈[0.001, 0.01, 0.1, 1, 10, 100] were tested, and the best parameters were penalty = ‘l1’ and C = 1. For the random forest model, the combinations of the number of estimators∈[50, 100, 150, 200, 250, 300, 500], maximum depth∈[20, 25, 30, 35, 40], and minimum samples in a leaf∈[1, 10, 20, 50, 100] were tested, and the best parameters were number of estimators = 250, maximum depth = 25, and minimum samples in a leaf = 10. For the SVM model, the combinations of C∈[0.001, 0.01, 0.1, 1, 10, 100] and gamma∈[0.001, 0.01, 0.1, 1, ‘auto’] were tested, and the best parameters were C = 10 and gamma = 0.01. For the neural network model, we built four models that each contain different numbers of hidden layers (one, two, three, and four hidden layers, respectively) with ReLU as activation functions for hidden units. Back-propagation was conducted using the Adam optimizer with a learning rate of 0.0001. Among four neural network models, we selected the model with three hidden layers as a representative neural network because its cross-validated AUC was better than that of others (S3 Table).

### Performance evaluation

Sensitivity, specificity, positive predictive value (PPV), negative predictive value (NPV), F-score, and area under receive operating characteristic curve (AUROC) were used as performance indexes. We applied 100 models for each ML algorithm generated through 10 experiments with tenfold cross-validation to the test dataset to evaluate the general performance of the ML models. We measured the variable importance using the Gini index as an impurity function in the random forest model and the magnitude of coefficient in the L1 regularized logistic regression model to assess the features that are relatively important to ADR detection.

To compare the performance of our models with that of other methods, we computed the performance of the CERT, CLEAR, and PACE algorithms and a simple combination of these methods as well as of the main measures used for signal detection in pharmacovigilance, including proportional ADR reporting ratio (PRR), reporting odds ratio (ROR), Yule’s Q (YULE), χ^2^ test (CHI), Bayesian confidence propagation neural network (BCPNN), and gamma Poisson shrinkage (GPS). For these algorithms (not ML models), the performance was evaluated on the entire dataset using their own criteria as used in previous studies: (1) for CERT, the *p*-value from the paired *t*-test or Wilcoxon’s signed-rank test (p < 0.05) or *p*-value from McNemar’s test or McNemar’s exact test (p < 0.05) among the pairs, in which more than 400 cases were used [19], and the result obtained according to these criteria was called “CERT400”; (2) for CLEAR, OR (>1) and the *p*-value from the conditional logistic regression (p < 0.05) were used [8], and the result was called “CLEAR”; (3) for PACE, we used the PCI (<0.667), and the result was called “PACE”; (4) the condition that one of CERT or CLEAR should be fulfilled and PCI < 0.667 was used, and the result was called “CCP2”; (5) the condition that CERT, CLEAR, and PACE should all be satisfied was used, and the result was called “CCP3”; and (6) for PRR, ROR, YULE, CHI, BCPNN, and GPS, PRR − 1.96SE > 1, ROR − 1.96SE > 1, YULE– 1.96SE > 1, CHI’s p < 0.05, information component (IC)—2SD > 0, and lower limit of Empirical Bayesian Geometric Mean 90% confidence interval (EB05) > 2 were used, respectively.

A one-way analysis of variance (ANOVA) followed by Tukey’s honestly significant difference (HSD) test were performed to compare and determine the significant differences between AUROCs among ML models.

### Software tools

MS-SQL 2017 was used for data management. Python (version 3.6.1) with scikit-learn[20] and TensorFlow[21] libraries were used to develop the ML models.

## Results

Table 2 summarizes the performance evaluation results for ML models and other models. Performance indexes of ML models outperformed those of the other algorithms by a large margin, indicating that ML models produced higher averaged F1-measures and AUROC (0.629–0.709 and 0.737–0.816 respectively) compared to those of the original methods (0.020–0.597 and 0.475–0.563, respectively).

**Table 2:** Performance of ML models and previous ADR signal detection methods.

	Criterion for signaling	Sensitivity	Specificity	PPV	NPV	F1-measure	AUROC
RF^†^	Probability > 0.5	0.671 (±0.054)	0.780 (±0.046)	0.727 (±0.050)	0.732 (±0.043)	0.696 (±0.041)	0.816 (±0.031)
SVM^†^	Probability > 0.5	0.569 (±0.056)	0.796 (±0.046)	0.709 (±0.053)	0.680 (±0.043)	0.629 (±0.045)	0.737 (±0.040)
L1LR ^†^	Probability > 0.5	0.593 (±0.063)	0.756 (±0.047)	0.679 (±0.048)	0.682 (±0.049)	0.631 (±0.047)	0.741 (±0.041)
NN^†^	Probability > 0.5	0.793 (±0.062)	0.619 (±0.061)	0.645 (±0.047)	0.777 (±0.052)	0.709 (±0.037)	0.795 (±0.034)
CEART400^‡^	p < 0.05 # of patients > 400	0.868	0.100	0.455	0.467	0.597	0.559
CLEAR^‡^	p < 0.05, OR > 1	0.674	0.413	0.496	0.596	0.571	0.559
PACE^‡^	PCI < 0.667	0.081	0.897	0.406	0.529	0.135	0.520
CCP2^‡^	(CERT: p < 0.05 or CLEAR: p < 0.05, OR > 1) PACE: PCI < 0.667	0.075	0.908	0.405	0.540	0.127	0.518
CCP3^‡^	CERT: p < 0.05 CLEAR: p < 0.05, OR > 1 ,PACE: PCI < 0.667	0.074	0.920	0.453	0.526	0.127	0.475
CHI^‡^	p < 0.05	0.486	0.517	0.466	0.537	0.476	0.563
PRR^‡^	PRR-1.96SE > 1	0.463	0.573	0.485	0.551	0.473	0.525
ROR^‡^	ROR-1.96SE > 1	0.563	0.483	0.486	0.560	0.522	0.563
YULE^‡^	Yule’s Q-1.96SE > 1	0.350	0.680	0.487	0.546	0.407	0.522
BCPNN^‡^	IC-2SD >0	0.508	0.521	0.479	0.549	0.493	0.517
GPS^‡^	EB05>2	0.010	1	1	0.538	0.020	0.524

RF, random forest; SVM, support vector machine; L1LR, L1 regularized logistic regression; NN, neural network with three hidden layers; CCP2, PCI is less than 0.667 and one of the criteria of CERT and CLEAR is fulfilled; CCP3, PCI is less than 0.667 and all criteria of CERT and CLEAR are fulfilled; CHI, χ^2^ test; PRR, proportional reporting ratios; ROR, reporting odds ratio; YULE, Yule’s Q; BCPNN, Bayesian Confidence Neural Network; GPS, gamma Poisson shrinker; PPV, positive predictive value; and NPV, negative predictive value

^†^Average ± standard deviation of the performance results from 10 experiments with tenfold cross-validation

^‡^Performance results on the whole dataset using their own criteria

**Fig 2:**
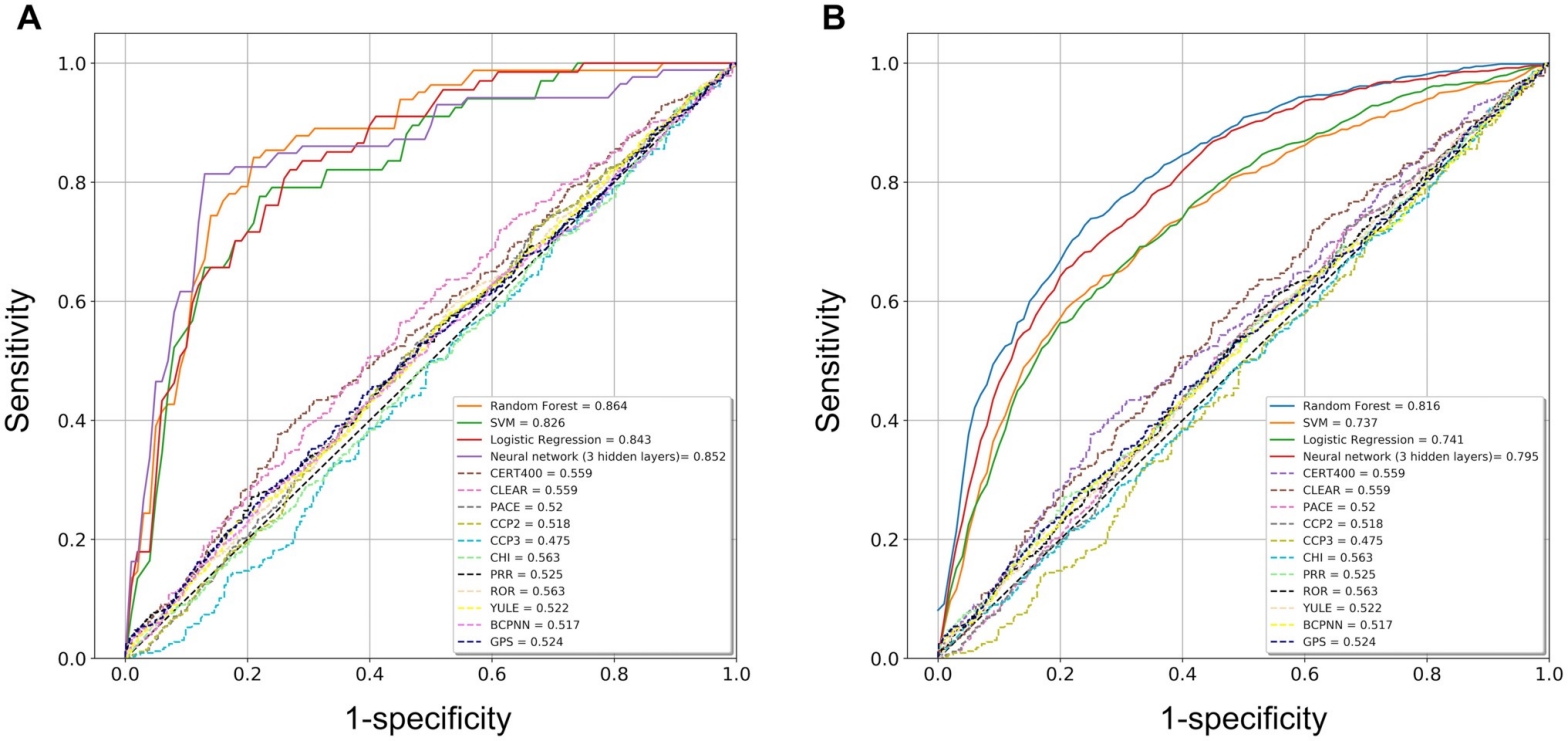
AUROCs of ML algorithms and original ADR signal detection algorithms. The best AUROCs (A) and average AUROCs (B) of each algorithm are shown among 10 experiments with tenfold cross-validation. No significant difference is observed in the ML models; however, the AUROCs of the ML models are much larger than those of the original methods.

Among all constructed models, neural network models had the highest sensitivity, NPV, and F1-measures, whereas random forest models had the highest PPV and SVM models had the highest specificity. When we compared the AUROCs of the ML models using ANOVA followed by Tukey’s HSD test, we found that the random forest models had significantly higher AUROCs on average than other models (p < 0.01, Table 3). The AUROC of the original methods using the whole dataset based on the original criteria was notably lower than that of the ML models (Fig 2).

**Table 3:** Summary table of Tukey’s HSD post-hoc test results among ML models.

(I) ML model	(J) ML model	Mean difference (I – J)	Std. error	Sig.	95% Confidence interval	
					Lower bound	Upper bound
NN	RF	-0.024	0.005	<0.01	-0.037	-0.010
	SVM	0.055	0.005	<0.01	0.042	0.069
	L1LR	0.051	0.005	<0.01	0.038	0.064
RF	NN	0.024	0.005	<0.01	0.010	0.037
	SVM	0.040	0.005	<0.01	0.026	0.054
	L1LR	0.075	0.005	<0.01	0.061	0.088
SVM	NN	-0.055	0.005	<0.01	-0.069	-0.042
	RF	-0.040	0.005	<0.01	-0.054	-0.026
	L1LR	-0.004	0.005	0.860	-0.017	0.009
L1LR	NN	-0.050	0.005	<0.01	-0.064	-0.038
	RF	-0.075	0.005	<0.01	-0.088	-0.061
	SVM	0.005	0.005	0.860	-0.009	0.017

**Fig 3:**
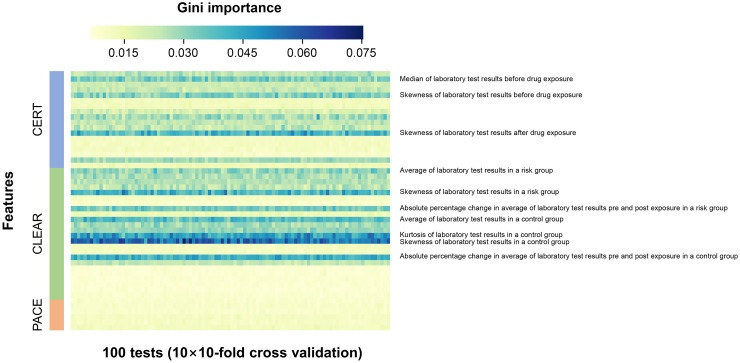
Important features in random forest algorithm. The importance of features is expressed in terms of Gini importance by color during 10 experiments with tenfold cross-validation. The blue color implies more importance and the yellow color, less importance. The top 10 important features are marked by red boxes.

**Fig 4:**
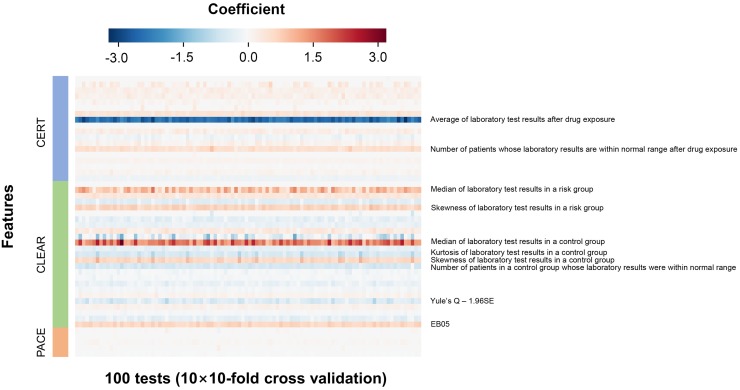
Coefficients calculated in logistic regression. The coefficients of features are expressed in color during 10 experiments with tenfold cross-validation. Red color indicates positive coefficients and blue color, negative coefficients.

To explore the effectiveness of features, we visualized and listed important features from our random forest and L1 regularized logistic regression models during 100 experiments with 10 experiments with tenfold cross-validation (Figs 3 and 4). The top 10 features considered important by the random forest classifier consisted of three features from the CERT algorithm and seven features from the CLEAR algorithm and were mainly related to the shape of the distribution, such as kurtosis or skewness, and descriptive statistics such as absolute percentage change, median, and average of laboratory test results, whereas the top 10 absolute values of coefficients from L1 regularized logistic regression included not only features related to the shape of distribution and the descriptive values but also features related to disproportionality analysis measures (Yule’s Q and EB05) from CLEAR algorithms. S4 Table summarizes feature importance scores and ranks of input features.

## Discussion

This study presented AI-based models to identify the ADR signals by combining features from three different ADR detection signal algorithms based on laboratory test results from inpatient EHR data. When analyzing the importance level of each feature, we found that the three algorithms compensated each other and contributed to improving the overall performance.

Since the publication of the original version of the three algorithms used in this study (i.e., CERT, CLEAR, and PACE), various upgraded algorithms have been developed and suggested by several researchers worldwide in recent years.[9, 11, 19] Tham et al. suggested that at least 400 cases should be analyzed to guarantee minimum performance when using the CERT algorithm.[19] This method is called CERT400. Liu et al. proposed the possibility of using methods that were traditionally used in the spontaneous reporting system (SRS), like ROR, instead of the conditional logistic regression when using the CLEAR algorithm.[9] Lee et al. applied the CLEAR algorithm to data for each year to calibrate yearly characteristics and performed a meta-analysis to obtain the final results.[11]

Our study differs from existing approaches. Previous studies attempted to develop a new algorithm to overcome the limitations of existing approaches. However, we found that none of them completely replaced the other; therefore, we developed a method in which the algorithms complement each other to provide results. Consequently, the ML models showed better detection performance compared to existing individual studies.

The results of the ML models that used the features of different previous algorithms were much better than those of individual previous algorithms or combined final outputs of each algorithm (CCP2 or CCP3). CLEAR showed sensitivity of 0.61 when evaluated in previous studies[9]; this was similar to the result obtained in our study (0.674). Similarly, CERT400 showed F1-measure of 0.598 when evaluated in previous studies; this was similar to the result obtained in our study (0.597). The PACE algorithm had very low sensitivity or PPV because its primary purpose was not ADR signal detection; however, its specificity was as high as 0.897 because its purpose is to filter out non-ADR signals. Therefore, we did not consider the performance of existing algorithms to be underestimated herein. Nonetheless, these results showed an improvement in performance and demonstrated the synergic effect of combining ML models.

The first reason for this might be that we did not simply synthesize the final results of each algorithm but the descriptive statistics and measures of shape that each algorithm used before making the final decision. Although each descriptive statistic and measure of shape was not primarily used in the evaluation of ADRs in previous algorithms, we believed that a meaningful correlation might exist in the real world. For example, features mainly related to the shape of distribution, such as kurtosis or skewness, were important factors in both random forest and L1 logistic regression models. In the L1 logistic regression model, ADR and kurtosis have a negative correlation and skewness has a positive correlation. It might suggest that the skewness of the laboratory test result distribution increased owing to the increase in extreme values caused by drugs and that kurtosis was decreased accordingly by the decreased frequency of normal values usually located at the center of the distribution.

The second reason might be that complex confounding effects exist in the EHR data.[22] Therefore, an algorithm alone will not be able to perfectly correct it, and algorithms that complement each other will be necessary. The main selected features showed that both CLEAR- and CERT-based features were simultaneously used for ADR signal detection, as shown in Figs 3 and 4.

The performance of each ML algorithm showed a slight difference with different performance indexes. For example, sensitivity was the highest at 0.793 for a neural network but the lowest at 0.645 for PPV. PPV was the highest at 0.727 in the random forest model, and sensitivity was the highest at 0.796 in SVM. A wide range of opinions exists on the index to be used when evaluating the performance of the ADR signal detection algorithm. In our study, as in general ML studies, AUROC or F1 scores were used for comprehensive evaluation. However, calculating the actual sensitivity or specificity is controversial because defining a true gold standard set for ADRs is unrealistic. Therefore, the list of known or unknown drug–laboratory event pairs is called the ADR reference dataset rather than the gold standard. In addition, PPV was used as an important performance factor in many previous articles.[8, 19] However, some studies showed that a high-sensitivity algorithm minimizes the signal detection time even if the PPV is low.[23]

We investigated the previously unknown drug–laboratory event pairs predicted to have a possible association with drug and ADR using all four ML algorithms. An examination of these pairs showed that several studies supported their association (S5 Table). For example, ML models in the study predicted that Candesartan and Irbesartan cause an increase in alkaline phosphatase level. This increase can usually be observed in hepatitis or biliary obstruction. Several recent case reports also suggest that Candesartan and Irbesartan is associated with drug-induced hepatitis, especially cholestatic hepatitis.[24–26] The presence of many referable drug–laboratory event correlations in unknown pairs, which our models predicted, indicates that our models can be used to provide reliable intimations for further investigations.

The prioritization of a signal after its detection is also important; however, this is an entirely manual task.[27] The results may differ depending on the individual’s experience or knowledge; thus, prioritization should be based on a variety of evidence. However, no definitive guide has been provided for decision-making. Therefore, we believe that ML models can be applied to prioritization in the future, as in this study.

Several studies have recently reported various successful phenotyping approaches with EHR data.[28] Therefore, the scope of future surveillance will be extended to various phenotypes as well as laboratory test results. For example, diabetes mellitus or dementia cannot be represented by a single laboratory test event. For detecting these ADR signals, they need to be phenotyped with EHR before applying ADR signal detection algorithms.

This study has limitations. First, data from only a single institute were used; therefore, only 1,674 drug–laboratory event pairs were available for evaluation despite many other possible combinations. Further validation study is needed using EHR data from different hospitals with different laboratory test procedures, practice patterns, and patient compositions. Second, the ADR reference standard cannot be perfect; however, we believe that the performance evaluation in our study was more objective than those in previous studies because the analysis was conducted on fairly large and diverse drug-laboratory event pairs compared to previous studies that used only used 4–500 selected pairs for specific drugs. Third, the results of this study may suggest but not confirm the possibility of a causal relationship. To confirm such a relationship, well-designed epidemiologic studies or methods for evaluating causal relationships, such as the Naranjo algorithm[29], on individual drug–laboratory event pairs are required.

## Conclusions

In this study, we built ML models to detect ADR signals from EHR data by consolidating the features from existing ADR signal detection algorithms. ML models showed better performance for ADR signal detection compared to previously proposed algorithms, suggesting that they can be useful tools for use in uncovering the possibility of adverse drug event associations for pharmacovigilance.

## Supporting information

S1 Table A mapping of drug names from ADR databases to RxNorm. (XLSX)

S2 Table All feature values of drug–laboratory event pairs, excluding pairs containing missing values. (XLSX)

S3 Table A comparison of the performance of neural network models with different numbers of hidden layers. (DOCX)

S4 Table Feature importance scores evaluated using random forest and logistic regression models. (XLSX)

S5 Table List of previously unknown drug–laboratory event pairs predicted to have a possible association by four ML algorithms and related studies that support their association. (DOCX)
