# Correction: Machine learning model combining features from algorithms with different analytical methodologies to detect laboratory-event-related adverse drug reaction signals

**DOI:** 10.1371/journal.pone.0215344

**Published:** 2019-04-09

**Authors:** Eugene Jeong, Namgi Park, Young Choi, Rae Woong Park, Dukyong Yoon

There is an error in the Funding statement. The correct Funding statement is as follows: This research was supported by grants from the Korea Health Technology R&D Project through the Korea Health Industry Development Institute (KHIDI) funded by the Ministry of Health & Welfare, Republic of Korea (grant numbers: HI16C0982 and HI17C0970, Government-wide R&D Fund project for infectious disease research, HG18C0067). The funder had no role in study design, data collection and analysis, decision to publish, or preparation of the manuscript.
