# Supplementary material for: Machine learning model combining features from algorithms with different analytical methodologies to detect laboratory-event-related adverse drug reaction signals
Source: PLoS One. 2018 Nov 21;13(11):e0207749. doi: 10.1371/journal.pone.0207749 (PMC6248973; doi:10.1371/journal.pone.0207749)
Supplement: S3 Table — (DOCX) [file pone.0207749.s003.docx]

**S3 Table. A comparison of the performance of the neural network models with different number of hidden layers.**

|  | # of neurons | Criterion for signaling | Sensitivity | Specificity | PPV | NPV | F1-measure | AUROC |
| --- | --- | --- | --- | --- | --- | --- | --- | --- |
| NN1^†^ | 1^st^ hidden layer: 48 | Probability > 0.5 | 0.567  (±0.069) | 0.798  (±0.052) | 0.711  (±0.054) | 0.680  (±0.044) | 0.628  (±0.048) | 0.751  (±0.034) |
| NN2^†^ | 1^st^ hidden layer: 48  2^nd^ hidden layer: 128 | Probability > 0.5 | 0.809  (±0.051) | 0.588  (±0.044) | 0.630  (±0.039) | 0.782  (±0.049) | 0.707  (±0.033) | 0.789  (±0.034) |
| NN3^†^ | 1^st^ hidden layer: 48  2^nd^ hidden layer: 128  3^rd^ hidden layer: 128 | Probability > 0.5 | 0.793  (±0.062) | 0.619  (±0.061) | 0.645  (±0.047) | 0.777  (±0.052) | 0.709  (±0.037) | 0.795  (±0.034) |
| NN4^†^ | 1^st^ hidden layer: 48  2^nd^ hidden layer: 128  3^rd^ hidden layer: 128  4^th^ hidden layer: 128 | Probability > 0.5 | 0.780  (±0.068) | 0.628  (±0.068) | 0.647  (±0.046) | 0.770  (±0.052) | 0.745  (±0.036) | 0.793  (±0.033) |

NN1, neural network with 1 hidden layer; NN2, neural network with 2 hidden layers; NN3, neural network with 3 hidden layers; NN4, neural network with 4 hidden layers

^†^Average ± standard deviation of the performance results from 10 experiments with a tenfold cross-validation
