# Supplementary material for: Machine learning model combining features from algorithms with different analytical methodologies to detect laboratory-event-related adverse drug reaction signals
Source: PLoS One. 2018 Nov 21;13(11):e0207749. doi: 10.1371/journal.pone.0207749 (PMC6248973; doi:10.1371/journal.pone.0207749)
Supplement: S5 Table — (DOCX) [file pone.0207749.s005.docx]

**S5 Table.** **List of the previously unknown drug–laboratory event pairs predicted to have a possible association with four ML algorithms and related studies that support their association**

| Drug | Laboratory test name | Type of abnormality | Reference(s) |
| --- | --- | --- | --- |
| Acetaminophen | Ferritin | Increase | [1, 2] |
| Acyclovir | Lipase | Increase |  |
| Arginine | T.Bilirubin | Increase |  |
| Bisoprolol | AST | Increase |  |
| Budesonide | Amylase | Increase |  |
| Candesartan | Alk.Phosphatase | Increase | [3, 4] |
| Budesonide | ALT | Increase |  |
| Cefdinir | ALT | Increase |  |
| Cefotetan | Alk.Phosphatase | Increase |  |
| Ceftizoxime | Lipase | Increase |  |
| Cefoxitin | AST | Increase |  |
| Cisplatin | Alk.Phosphatase | Increase | [5] |
| Citric Acid | Creatinine | Increase |  |
| Cyclosporine | T.Bilirubin | Increase |  |
| desmopressin | Creatinine | Increase |  |
| Dolasetron | D.Bilirubin | Increase |  |
| Doxorubicin | D.Bilirubin | Increase | [6, 7] |
| Doxazosin | T.Bilirubin | Increase |  |
| Doxazosin | AST | Increase |  |
| Enalapril | AST | Increase | [8, 9] |
| Esomeprazole | Creatinine | Increase |  |
| fesoterodine | AST | Increase |  |
| Fluoxetine | Creatinine | Increase |  |
| Fluconazole | Hemoglobin | Decrease | [10] |
| Flurbiprofen | Creatinine | Increase | [11, 12] |
| Gabapentin | T.Bilirubin | Increase | [13, 14] |
| Furosemide | Alk.Phosphatase | Increase | [15] |
| Ganciclovir | CK-MB | Increase |  |
| Irbesartan | AST | Increase | [16, 17] |
| Itraconazole | T.Bilirubin | Increase | [18, 19] |
| Irbesartan | T.Bilirubin | Increase | [16, 17] |
| Levodopa | CK-MB | Increase |  |
| Nimodipine | CK-MB | Increase |  |
| Nateglinide | Creatine Kinase | Increase |  |
| Pamidronate | Hemoglobin | Decrease |  |
| Piperacillin | Lactate Dehydrogenase | Increase | [20, 21] |
| Piperacillin | Creatine Kinase | Increase |  |
| Paroxetine | Creatinine | Increase | [22] |
| Ranitidine | Lactate Dehydrogenase | Increase | [23] |
| Rifampin | Creatine Kinase | Increase | [24, 25] |
| Rifampin | AST | Increase | [26, 27] |
| Rifampin | ALT | Increase | [26, 27] |
| Sorafenib | Creatinine | Increase | [28] |
| Tazobactam | T.Bilirubin | Increase | [29] |
| Tenofovir disoproxil | ALT | Decrease | ^[30]*^ |
| Tobramycin | Hemoglobin | Decrease |  |
| Theophylline | Creatinine | Increase |  |
| Valproate | Troponin I | Increase |  |

AST, aspartate aminotransferase; ALT, alanine aminotransferase; and CK-MB, creatine kinase-MB

^*^Effect of drug rather than adverse reaction

**References**

1. Eastham EJ, Bell JI, Douglas AP. Serum ferritin levels in acute hepatocellular damage from paracetamol overdosage. Br Med J. 1976;1(6012):750-1. Epub 1976/03/27. PubMed PMID: 1260314; PubMed Central PMCID: PMCPMC1639240.

2. Kotoh K, Ueda A, Tanaka M, Miyazaki M, Kato M, Kohjima M, et al. A high prevalence of extreme hyperferritinemia in acute hepatitis patients. Hepat Med. 2009;1:1-8. Epub 2009/01/01. PubMed PMID: 24623996; PubMed Central PMCID: PMCPMC3921818.

3. Jimenez-Saenz M, Arroyo Q, Sanjuan M, Herrerias JM. [Candesartan-induced cholestatic hepatitis: a case report]. Gastroenterol Hepatol. 2010;33(1):66-7. doi: 10.1016/j.gastrohep.2009.06.003. PubMed PMID: 19713003.

4. Vallejo I, Garcia Morillo S, Pamies E. [Acute hepatitis induced by candesartan]. Med Clin (Barc). 2000;115(18):719. PubMed PMID: 11141435.

5. Li X, Pan E, Zhu J, Xu L, Chen X, Li J, et al. Cisplatin Enhances Hepatitis B Virus Replication and PGC-1alpha Expression through Endoplasmic Reticulum Stress. Sci Rep. 2018;8(1):3496. doi: 10.1038/s41598-018-21847-3. PubMed PMID: 29472690; PubMed Central PMCID: PMCPMC5823916.

6. Chen YF, Chong CL, Wu YC, Wang YL, Tsai KN, Kuo TM, et al. Doxorubicin Activates Hepatitis B Virus Replication by Elevation of p21 (Waf1/Cip1) and C/EBPalpha Expression. PLoS One. 2015;10(6):e0131743. doi: 10.1371/journal.pone.0131743. PubMed PMID: 26121644; PubMed Central PMCID: PMCPMC4486450.

7. Wang J, Jia J, Chen R, Ding S, Xu Q, Zhang T, et al. RFX1 participates in doxorubicin-induced hepatitis B virus reactivation. Cancer Med. 2018;7(5):2021-33. doi: 10.1002/cam4.1468. PubMed PMID: 29601674; PubMed Central PMCID: PMCPMC5943424.

8. Bas V, Erkan T, Caliskan S, Sever L, Kasapcopur O, Ozbay G, et al. Toxic hepatitis due to enalapril in childhood. Pediatr Int. 2003;45(6):755-7. PubMed PMID: 14651558.

9. Hurlimann R, Binek J, Oehlschlegel C, Hammer B. [Enalapril (Reniten)-associated toxic hepatitis]. Schweiz Med Wochenschr. 1994;124(29):1276-80. PubMed PMID: 8066414.

10. Rodrigues JR, Lourenco D, Gamboa N. Disturbance in hemoglobin metabolism and in vivo antimalarial activity of azole antimycotics. Rev Inst Med Trop Sao Paulo. 2011;53(1):25-9. PubMed PMID: 21412616.

11. Banal F, Lecoules S, Carmoi T, Thomas L, Bonnefoy S, Bechade D, et al. [Acute renal insufficiency after flurbiprofen treatment in a patient treated with angiotensin converting enzyme inhibitor]. Rev Med Interne. 2008;29(7):593-5. doi: 10.1016/j.revmed.2007.12.014. PubMed PMID: 18280012.

12. MacKay K. Membranous nephropathy associated with the use of flurbiprofen. Clin Nephrol. 1997;47(4):279-80. PubMed PMID: 9128803.

13. Fuzier R, Serres I, Guitton E, Lapeyre-Mestre M, Montastruc JL, French Network of Pharmacovigilance C. Adverse drug reactions to gabapentin and pregabalin: a review of the French pharmacovigilance database. Drug Saf. 2013;36(1):55-62. doi: 10.1007/s40264-012-0006-6. PubMed PMID: 23315296.

14. Bureau C, Poirson H, Peron JM, Vinel JP. [Gabapentine-induced acute hepatitis]. Gastroenterol Clin Biol. 2003;27(12):1169-70. PubMed PMID: 14770125.

15. Pizzarossa AC, Rebella M. Hypoxic hepatitis and furosemide. BMJ Case Rep. 2018;2018. doi: 10.1136/bcr-2018-225497. PubMed PMID: 30275022.

16. Annicchiarico BE, Siciliano M. Could irbesartan trigger autoimmune cholestatic hepatitis? Eur J Gastroenterol Hepatol. 2005;17(2):247-8. PubMed PMID: 15674105.

17. Andrade RJ, Lucena MI, Fernandez MC, Vega JL, Garcia-Cortes M, Casado M, et al. Cholestatic hepatitis related to use of irbesartan: a case report and a literature review of angiotensin II antagonist-associated hepatotoxicity. Eur J Gastroenterol Hepatol. 2002;14(8):887-90. PubMed PMID: 12172412.

18. Lou HY, Fang CL, Fang SU, Tiong C, Cheng YC, Chang CC. Hepatic failure related to itraconazole use successfully treated by corticosteroids. Hepat Mon. 2011;11(10):843-6. doi: 10.5812/kowsar.1735143X.755. PubMed PMID: 22224084; PubMed Central PMCID: PMCPMC3234573.

19. Tuccori M, Bresci F, Guidi B, Blandizzi C, Del Tacca M, Di Paolo M. Fatal hepatitis after long-term pulse itraconazole treatment for onychomycosis. Ann Pharmacother. 2008;42(7):1112-7. doi: 10.1345/aph.1L051. PubMed PMID: 18523232.

20. Kerkhoff AD, Patrick L, Cornett P, Kleinhenz ME, Brondfield S. Severe piperacillin-tazobactam-induced hemolysis in a cystic fibrosis patient. Clin Case Rep. 2017;5(12):2059-61. doi: 10.1002/ccr3.1256. PubMed PMID: 29225856; PubMed Central PMCID: PMCPMC5715418.

21. Zanetti RC, Biswas AK. Hemolytic anemia as a result of piperacillin/tazobactam administration: a case report and discussion of pathophysiology. Mil Med. 2013;178(9):e1045-7. doi: 10.7205/MILMED-D-12-00512. PubMed PMID: 24005557.

22. Chen WC, Huang CC, Huang CJ, Chien JM, Lin KL, Lu YC, et al. Mechanism of paroxetine-induced cell death in renal tubular cells. Basic Clin Pharmacol Toxicol. 2008;103(5):407-13. doi: 10.1111/j.1742-7843.2008.00319.x. PubMed PMID: 18801027.

23. Takimoto R, Mogi Y, Kura T, Niitsu Y. [Hemolytic anemia and thrombocytopenia induced by cimetidine: recurrence with ranitidine administration]. Rinsho Ketsueki. 1997;38(2):124-8. PubMed PMID: 9059066.

24. Pogue JM, Lee J, Marchaim D, Yee V, Zhao JJ, Chopra T, et al. Incidence of and risk factors for colistin-associated nephrotoxicity in a large academic health system. Clin Infect Dis. 2011;53(9):879-84. doi: 10.1093/cid/cir611. PubMed PMID: 21900484.

25. Kumar BD, Prasad CE, Krishnaswamy K. Detection of rifampicin-induced nephrotoxicity by N-acetyl-3-D-glucosaminidase activity. J Trop Med Hyg. 1992;95(6):424-7. PubMed PMID: 1460703.

26. Bright-Thomas RJ, Gondker AR, Morris J, Ormerod LP. Drug-related hepatitis in patients treated with standard anti-tuberculosis chemotherapy over a 30-year period. Int J Tuberc Lung Dis. 2016;20(12):1621-4. PubMed PMID: 27931337.

27. Chogtu B, Surendra VU, Magazine R, Acharya PR, Yerrapragada DB. Rifampicin-Induced Concomitant Renal Injury and Hepatitis. J Clin Diagn Res. 2016;10(9):OD18-OD9. doi: 10.7860/JCDR/2016/21030.8578. PubMed PMID: 27790502; PubMed Central PMCID: PMCPMC5072002.

28. Rollino C, Beltrame G, Ferro M, Quattrocchio G, Tonda L, Quarello F. [Cancer treatment-induced nephrotoxicity: BCR-Abl and VEGF inhibitors]. G Ital Nefrol. 2010;27 Suppl 50:S70-4. PubMed PMID: 20922699.

29. Kraleti S, Khatri N, Jarrett D. Piperacillin-Tazobactam Induced Interstitial Nephritis, Hepatitis and Serum Sckness-Like Illness. J Ark Med Soc. 2016;112(14):278-80. PubMed PMID: 27434982.

30. Murray KF, Szenborn L, Wysocki J, Rossi S, Corsa AC, Dinh P, et al. Randomized, placebo-controlled trial of tenofovir disoproxil fumarate in adolescents with chronic hepatitis B. Hepatology. 2012;56(6):2018-26. doi: 10.1002/hep.25818. PubMed PMID: 22544804.
